# A Journey in Science: Molecular vaccines for global child health in troubled times of anti-science

**DOI:** 10.1186/s10020-024-00786-y

**Published:** 2024-03-15

**Authors:** Peter J. Hotez

**Affiliations:** Texas Children’s Hospital Center for Vaccine Development, Departments of Pediatrics and Molecular Virology, National School of Tropical Medicine, Baylor College of Medicine, Houston, TX 77030 USA

## Abstract

My scientific life in translational medicine runs in two parallel, yet often converging paths. The first, is four-decade-long commitment to develop new vaccines for parasitic and neglected tropical diseases, as well as pandemic threats. This includes a vaccine for human hookworm infection that I began as an MD-PhD student in New York City in the 1980s, and a new low-cost COVID vaccine that reached almost 100 million people in low- and middle-income countries. Alongside this life in scientific research, is one in public engagement for vaccine and neglected disease diplomacy to ensure that people who live in extreme poverty can benefit from access to biomedical innovations. A troubling element has been the daunting task of countering rising antivaccine activism, which threatens to undermine our global vaccine ecosystem. Yet, this activity may turn out to become just as important for saving lives as developing new vaccines.

## Abstract (zh)

Real innovations in medicine and science are historic and singular; the stories behind each occurrence are precious. At Molecular Medicine we have established the Anthony Cerami Award in Translational Medicine to document and preserve these histories. The monographs recount the seminal events as told in the voice of the original investigators who provided the crucial early insight. These essays capture the essence of discovery, chronicling the birth of ideas that created new fields of research and launched trajectories that persisted and ultimately influenced how disease is prevented, diagnosed, and treated. In this volume, the Cerami Award Monograph is by Peter Hotez, MD, PhD, DSc (hon), FASTMH, FAAP, co-director of the Texas Children’s Hospital Center for Vaccine Development, the Texas Children’s Hospital Endowed Chair in Tropical Pediatrics, and professor and dean of the National School of Tropical Medicine at Baylor College of Medicine. A distinguished vaccinologist, pediatrician, and global health advocate with remarkable achievements in the realm of molecular medicine, this is the story of Dr. Hotez’s scientific journey.

## Introduction and overview

Ever since I can remember, I have admired scientists who use their knowledge for humanitarian pursuits. While one could argue that all science benefits humanity, and I mostly agree with this point, there are two specific scientific activities (and two types of scientists) that for me have special attraction. The first are biomedical scientists who devote their research to translational medicine in vaccinology; the second are scientists who use their knowledge (or stature) to step outside the laboratory and pursue some aspect of social justice or statesmanship—meaning diplomacy or public affairs for the common good.

The great vaccinologists have always been my role models. They include Drs. John Robbins and Rachel Schneerson who developed the *Haemophilus influenzae* type b (Hib) vaccine (Robbins and Schneerson 1987). Working at the National Institute of Child Health and Development (NICHD) of the US National Institutes of Health (NIH), the Hib vaccine developed by Robbins and Schneerson had an almost immediate public health impact. As a pediatric house officer in Boston at Massachusetts General Hospital from 1987 to 1989, I admitted and took care of many sick children with Hib meningitis. Many of those kids suffered deafness or other permanent neurologic injuries. Some died from their Hib meningitis. Then, the Robbins-Schneerson vaccine, as well as a similar vaccine developed by Drs. David Smith and Porter Anderson (Smith et al. 1989) began to enter widespread use and permeate pediatric practices in America. As a result, by the time I had finished my pediatrics residency and a subsequent pediatric infectious diseases fellowship at Yale University School of Medicine, I no longer routinely admitted sick children with Hib meningitis. Over the course of just a few years, Hib meningitis had mostly disappeared in the United States because of the development, production, and distribution of a vaccine. This achievement highlighted the power of a safe and effective vaccine. However, even before the Hib vaccine I had read about the impressive public health impact of vaccines for rabies, yellow fever, polio, measles, and many other infectious diseases, and aspired to become a vaccine scientist. I devoted my life to developing new vaccines for parasitic and tropical infections, a group now often referred to as the neglected tropical diseases. The human hookworm vaccine I began for my MD-PhD thesis in New York City at Rockefeller University is now in phase 2 clinical trials and showing promise in a human challenge model of hookworm infection (Puchner et al. 2023). Other vaccines followed including a coronavirus vaccine technology leading to almost 100 million vaccine doses administered to children and adults in India and Indonesia (Hotez et al. 2023).

In parallel, I have long-admired scientists who simultaneously pioneered an expanded use of vaccines to promote international peace, a concept I once named vaccine diplomacy (Hotez 2001). Vaccine diplomacy goes back to Dr. Edward Jenner who developed the first vaccine for smallpox at the end of the eighteenth century, but in more modern times, Dr. Albert Sabin developed the oral polio vaccine jointly with his Soviet Russian counterparts during some of the toughest years of the Cold War (Hotez 2014, 2021). I aspired to replicate this accomplishment in some twenty-first century form. Most of the vaccines our laboratory—the Texas Children’s Hospital Center for Vaccine Development—currently develops are advanced jointly with scientists in low- and middle-income countries (LMICs), including Brazil, India, Indonesia, Malaysia, and Mexico. I embarked on this modern version of vaccine diplomacy while serving as a United Science Envoy in the Obama Administration in 2015–2016 (Hotez 2021). It is a form of public engagement I cherish, and one that could one day help reinforce pandemic preparedness and promote global health.

For me, a more challenging aspect of vaccine diplomacy has been countering widening antivaccine activism. The idea of antivaccine activists or those who would seek to undermine the public confidence in vaccines was something I had not imagined as an MD-PhD student in the 1980s. However, now antivaccine activism is a powerful and globalizing force that causes thousands of people to shun their immunizations, including parents vaccinating their children against diseases such as Hib. My entry into the public square defending vaccines was not planned. This aspect of my life in science began after it was first alleged in late 1990s that vaccines such as the measles-mumps-rubella (MMR) live virus vaccine cause or contribute to autism. One of my four children, Rachel, has autism and intellectual disabilities, and I wrote a book with Johns Hopkins University Press—*Vaccines Did Not Cause Rachel’s Autism: My Journey as a Vaccine Scientist, Pediatrician, and Autism Dad*—which ultimately made me a prime target for antivaccine activists (Hotez 2018). Today, antivaccine activism has been embraced by major political parties in the United States and elsewhere. In America, many elected leaders or public officials now openly espouse antivaccine sentiments and some even openly discourage Americans from accepting vaccines as a safe and effective public health control tool. As an outspoken defender of vaccines in the public square, I have become a major target. This has expanded significance because now the antivaccine movement has unprecedented levels of political power, organization, and financial support (Hotez 2023a, b). My pointing out that 200,000 Americans needlessly perished during the COVID-19 pandemic because they refused COVID-19 immunizations infuriates antivaccine activist members of the United States Congress and at least two major Presidential candidates. Their adherents sometimes seek retribution. Even though I am threatened online or even stalked by antivaccine activists when I give public lectures, I feel that defending vaccines publicly has become almost as important in terms of saving lives as developing new vaccines. I therefore pursue two parallel, although frequently convergent, careers in science—as both a vaccinologist developing new vaccines for child global health and as an ardent public advocate of immunizations globally and in the United States.

## Early education

**Fig. 1:**
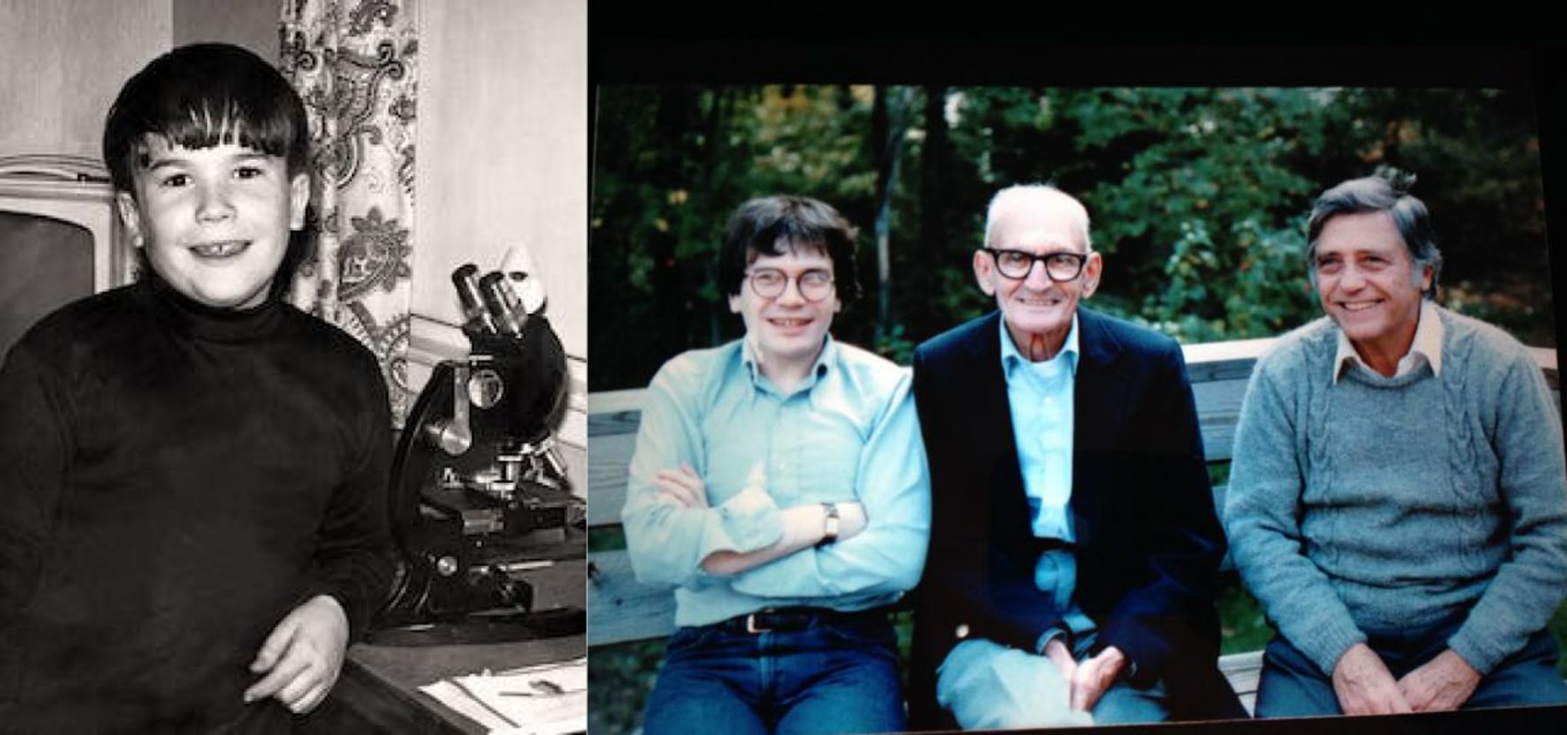
The author (left) with his microscope in West Hartford, Connecticut during the 1960s, and (right) seated immediately next to his grandfather, Morris Goldberg, and to his right, the author’s father, Eddie Hotez. Sometime in the late 1970s or early 1980s

I wanted to be a scientist studying microorganisms from a very early age. Figure 1 (left) shows me as a boy maybe around 10 years of age, very proud to be sitting with my microscope. I grew up in a solidly middle-class home in West Hartford, Connecticut, not far from a brook where I would collect water and search for protozoa, guided by my favorite book, *Hunting with the Microscope*, illustrated with no frills hand drawings (Johnson and Bleifield 1956). My childhood was both stable and safe, with two supportive parents, and grandparents on my mother’s side who lived nearby. Pictured on the right I’m seated with my grandfather, Morris Goldberg and my dad, Eddie Hotez.

All four of my grandparents were Jewish immigrants. My father’s parents were from a part of Western Ukraine, known as Galicia, where its inhabitants are sometimes known as Galitizianers. Galitzianers have a mixed reputation. According to some they are considered backwards or even hillbillies. However, the Jewish public intellectual and longtime *Commentary* editor, Norman Podhoretz, writes that Jewish intellectuals from Galicia were partly responsible for the Haskalah, the Jewish enlightenment (Podhoretz 2001). It is possible my Galitzianer roots are responsible for the letter Z in my name (like Podhoretz), although there are differences of opinion where the Hotez name originates.

On my mother’s side, my grandmother, Rose Krechevsky, was one of many children born into the same family in Belarus. Her brother, David, shortened to David Krech, became a famous psychology professor at the University of California Berkeley, where he spoke out on social justice issues including the harmful effects of segregation in public schools (Innis 1998). I sometimes wonder if my public positions defending vaccines or speaking out on behalf of people who live in poverty with neglected parasitic infections were somehow passed down from Uncle David. My grandfather Morris Goldberg, also known as Maurice or Moishe, was born in Leeds, England but grew up in the Jewish Quarter of Paris on Rue des Rosiers. His parents were actors in the Yiddish theatre but divorced. Eventually, the biological father took Morris to Montreal, Quebec before he made his way down to Hartford, Connecticut and met my grandmother, Rose. He was a bit of an adventurer, and I remember Morris telling me about the time he was stopped for speeding in Las Vegas, Nevada while in his 80 s.

I suppose that creative mix of public intellectual and street-smart immigrants helped to create the scientist I was to become. The Holocaust was another influence. Morris lost a sister he left behind in Paris and grieved for her until the end of his long life. I also had cousins on my mother’s side, Phil and Ruth Lazowski, who wrote about their harrowing story of escaping the Nazi’s and coming to America (Lazowski 2006; Frankel 2021). This background is a reason I have written about the dangers of antisemitism, and why it is so hurtful when I am attacked during the COVID-19 pandemic for being a Jewish vaccine scientist (Hotez 2023c, d).

My dad, Eddie Hotez, grew up in Bronx, New York, where he attended public schools. During World War II attended City College of New York, like many first-generation Jewish immigrants, and later Trinity College in Hartford, Connecticut after signing up for the Navy’s V-12 Officer Training Program. Eddie thought initially he had a promise to get rushed through medical school and become a Naval physician, but instead he was assigned to a Landing Ship Transport (LST) in the Pacific Theatre. My dad saw action in Saipan, Okinawa, and Philippines and told me stories about how he and his shipmates came under attack by Japanese fighter planes and zeros. After the war, he had met my mother Jean Goldberg and proceeded to start a family instead of attending medical school. At the age of 89, after a raising four children, including me, and having several grandchildren, Eddie was buried with full military honors in a Jewish cemetery in Avon, Connecticut in 2015. During his lifetime, Eddie made certain that all three sons would become physicians. I was the only physician-scientist, while my brother Richard Hotes MD (I never understood the name change from Hotez) became an anesthesiologist in Hartford, Connecticut, tragically passing away at the age of 49 with amyloidosis (something our family has never quite recovered from). My oldest brother, Lawrence Hotes MD, is a successful internist in Boston, serving as chief medical officers for hospitals connected to both Boston University and Tufts medical schools. My sister, Elizabeth Kirshenbaum became a successful lawyer and mental health counselor in Boston. My mother Jean Goldberg Hotez grew up in Hartford, Connecticut raising the family. She was a great beauty and sang on the radio. Her brother (my uncle) Irving Goldberg MD PhD was a brilliant physician-scientist and former pharmacology department chair at Harvard Medical School, while his son Daniel Goldberg MD PhD, my cousin and friend, is an important professor at Washington University St. Louis. Nancy Goldberg, also a cousin and friend, is a pediatric gastroenterology nurse practitioner at Boston Children’s Hospital.

I studied very hard in Junior High School and High School in West Hartford, Connecticut, while always maintaining an interest in microorganisms. I also remember from an early age being fascinated by maps. Perhaps this combination helps explain my passion for studying tropical infectious diseases. Some of my closest friends from Connecticut loved music and Eastern philosophy, including Joe Pava who passed away tragically too early at 62, and from them I have many cherished memories that activate almost anytime I hear an interesting piece of music or see books on Buddhism or Taoism in bookstores. As an adolescent, I can still remember reading a parasitology text in our public library. It was *Introduction to Parasitology* by Drs. Asa C. Chandler and Clark P. Read, so at a quite a young age I had that fascination. Later while still in Junior High School, I purchased my own copies of *Craig and Faust’s Clinical Parasitology* and *Manson’s Tropical Diseases*. I was also blessed to have a wonderful high school science teacher, Daniel Hoyt, who took me under his wing to do a Westinghouse Science project and because I lived just a few miles away from the University of Connecticut medical school, I volunteered on research projects in Dr. Robert Poyton’s laboratory in the Department of Microbiology, isolating mitochondria from yeast. That provided my first experience with high-speed centrifuges, gel electrophoresis, chromatographic columns, and many other techniques, which are so “bread and butter” to a modern molecular biology laboratory.

In 1976 I entered Yale University as freshman where I majored in Molecular Biophysics and Biochemistry. Aside from three extraordinary college roommates—Matthew Waldor, who became an important professor studying cholera at Harvard Medical School; Joseph Koerner, an important professor of art history at Harvard University; and Jim Silverblatt, a physician in practice in Ohio, my most memorable undergraduate experiences were working in two Yale laboratories in the medical school. One was headed by Prof. Curtis Patton PhD, who was one of the first African-American professors on the Yale basic science faculty, while the other was headed by Prof. Frank Richard MD, a British internist and physician-scientist who was a pioneer in studying the basic biochemistry and genetics of antibody diversity.

Both Curtis and Frank were important scientific influences and mentors. Before coming to Yale, Dr. Patton was a postdoctoral fellow in Professor Bill Trager’s laboratory (Rockefeller University) working on the biology of African trypanosomes. Curtis brought trypanosomes to Yale to start his laboratory as a faculty member. Frank also had a fascinating life history, most of which I only learned about much later in my scientific career. He was born in Berlin, Germany into an assimilated Jewish family, as Frank Sussmann, ultimately escaping with his family to England sometime between 1938 and 1939. This information is according to a detailed memoir written by Frank’s widow (also an important Yale scientist), Dr. Martine Armstrong. As a young adult in England, Frank now with a family name of Richards served in the Royal Air Force, before attending Cambridge University as an undergraduate and later graduating from St. Mary’s medical school in London. He became a physician-scientist studying porphyria and immigrated to the United States where he studied antibody diversity at Massachusetts General Hospital and later at Yale University School of Medicine.

As a Yale professor, Frank Richards became intrigued by the molecular genetic similarities between the generation of antibody diversity and the wide range of surface antigens produced by African trypanosomes, a flagellated parasitic protozoan and the cause of human and animal sleeping sickness.

The 1970s were an important time in the modern history of biomedicine. This is the period when the first eukaryotic genes were cloned (Morrow et al. 1974), and the Richards lab was among the first to apply modern molecular biology methods to the study of parasites. I spent almost all my free time in the Richards laboratory learning basic techniques around working with proteins and nucleic acids and purifying both from African trypanosomes. This was the beginning of new field of molecular parasitology. It is also when I published my first scientific paper as a co-author (Richards et al. 1981), with more to follow. I could not have been more thrilled and was committed to continuing in this important new field as an MD-PhD student after graduating from Yale in 1980.

## Embarking on a Hookworm vaccine: Rockefeller University and the Laboratory of Medical Biochemistry

The Rockefeller University, beginning as the Rockefeller Institute of Medical Research, in New York City has an important place in the twentieth century history of infectious diseases and molecular medicine. Its pivotal role in America’s infectious disease research enterprise was detailed on John M. Barry’s book *The Great Influenza: The Epic Story of the Deadliest Plague in History* (Barry 2004). For me, however, the most relevant was a commitment made in the 1980s by Rockefeller University’s leadership to apply its scientific horsepower to the study of parasitic infections affecting LMICs. Some of Rockefeller University’s most important professors including the Nobel Laureate, Prof. Christian de Duve, as well as Profs. Miklos Muller, Zanvil Cohn, and Anthony Cerami, made commitments to devote a part of their laboratory research to the study of medically important parasites. In addition, Prof. William Traeger’s Laboratory of Parasitology at Rockefeller University had just become the first scientific group to successfully cultivate malaria parasites in vitro, and shortly after I arrived, Prof. George A.M. Cross was recruited from the United Kingdom to lead an expanded initiative in the molecular biology of parasitic protozoa. During the 1980s and for the next two decades, Rockefeller University had become an epicenter for the new science of molecular parasitology. Another attractive feature about Rockefeller University was its commitment to translational medicine and using the knowledge gained in their laboratories to develop new drugs or vaccines. Indeed, the Rockefeller University Hospital was the first research hospital in the United States created expressly for translational medicine (Comer 1964).

As a Yale senior undergraduate I felt the newly established combined MD and PhD programs represented the best path to become a medical scientist, and for studying parasitic and tropical infections, the combined MD-PhD program at Cornell Medical College (now Weill Cornell Medical College) and Rockefeller University was the best. Rockefeller and Weill Cornell are located next to one another on the Upper East Side of New York City where they form a large life sciences complex. Today, the program is known as the Tri-Institutional MD-PhD Program because it also now includes Memorial Sloan Kettering Cancer Center. My MD-PhD interview that cold December 1979 day in Manhattan was the first time I met Prof. Anthony “Tony” Cerami (Keating 2021). I can still remember how thrilling it was to sit with him at my interview and the way he showed genuine interest in the scientific ideas of someone like me who was still a senior college undergraduate. It is a lesson I have never forgotten – take young people and their ideas seriously and treat them as much as possible like colleagues rather than subordinates or “students”. I try to do this in my laboratory and over the decades I have discovered that young scientists oftentimes have the most innovative or thoughtful ideas and projects.

I was over the moon when a few weeks later I received my formal acceptance to the Cornell-Rockefeller MD PhD program. Although the opportunity to pursue my dream of studying molecular parasitology was the most important aspect, there were other considerations. Many of the MD-PhD programs in the United States are funded through an NIH-supported Medical Scientist Training Program. This meant I could obtain my doctoral degrees without incurring any debt and even receive a modest living stipend. Also, the prospect of living in New York City was very exciting for me, and I was thrilled to be living in Manhattan.

My MD-PhD program, like most, began with two years of basic sciences in medical school before embarking on graduate school research. Something Tony and I did while I was still in medical school those first two years (and which I have never forgotten) is when we would meet for breakfast on Friday mornings to discuss potential doctoral dissertation topics. Tony had a unique philosophy for his MD-PhD students, namely that they should bring new projects to the laboratory and use it as a steppingstone for their careers. The MD-PhD students who both preceded and followed me in Tony’s laboratory each went on to distinguished careers as leaders in biomedicine including Drs. Ron Koenig (University of Michigan), Nina Tabachnik (now Nina Schor at the NIH), Steve Meshnick (University of North Carolina), Rick Bucala (Yale), Christina Luedke (Boston Children’s Hospital) and others. Also, Dr. Annette Lee (Feinstein Institutes of Medical Research) did her dissertation research with Tony Cerami at Rockefeller University, as did Alejanro Zentella-Dehesa, a distinguished researcher in Mexico. Kevin Tracey MD PhD, the Feinstein Institutes President and Chief Executive Officer (CEO) also worked extensively with Dr. Cerami, as did Bruce Beutler MD who went on to win the Nobel Prize for his work discovering the toll-like receptor. At that time, Tony had a major interest in understanding the toxic and systemic effects of cancer and sepsis and Bruce did important work identifying those factors. So many physician-scientists came through the Laboratory of Medical Biochemistry it is difficult to name them all. Needless to say, the Cerami laboratory was a meaningful experience that put me on lifelong path of intellectual inquiry.

**Fig. 2:**
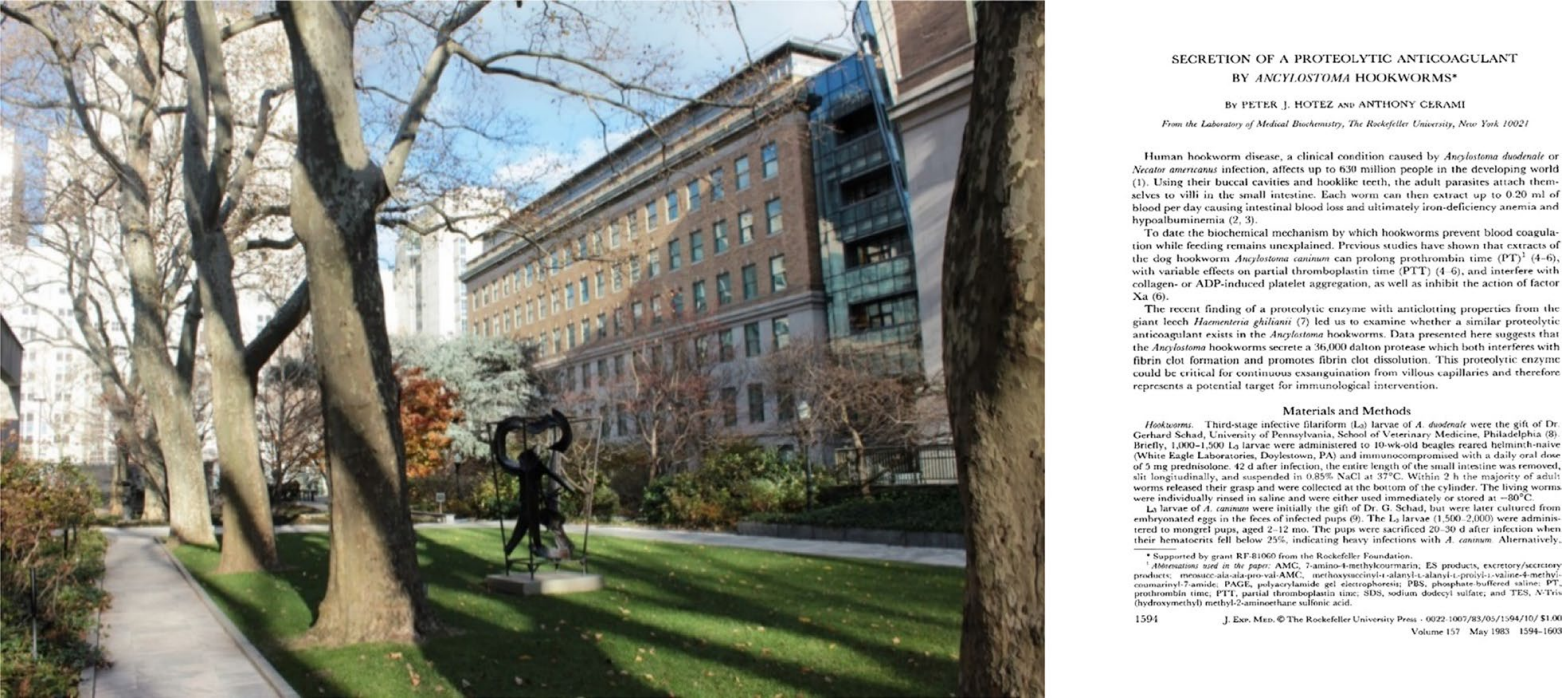
Left: Theobald Smith Hall, the initial location of the Laboratory of Medical Biochemistry (Cerami Laboratory), http://himetop.wikidot.com/smith-hall. Right: The author’s first, first author paper with Dr. Cerami in the *Journal of Experimental Medicine* (Hotez and Cerami 1983)

During the early 1980s, Tony’s laboratory—the Laboratory of Medical Biochemistry—was in the basement of Theobald Smith Hall (Fig. 2), named for a prominent late nineteenth century—early twentieth century microbiologist who was the first to discover how protozoan parasites causing Texas fever in cattle were transmitted by tick bites. This was one of the first demonstrations of arthropod-borne transmission of an infectious agent (Assadian and Stanek 2002).

In our weekly Friday meetings, we discussed multiple interesting projects, almost all of them related to the discovery of some new approach to therapeutics or vaccines for parasitic infections. During this period, the Laboratory of Medical Biochemistry received funding the Rockefeller Foundation. Under the director of Kenneth Warren MD, the Rockefeller Foundation had created a Great Neglected Diseases (GND) program to encourage modern molecular biology laboratories at prestigious institutions to take up the study of parasitic infections affecting LMICs (Keating 2017). The GND established an international network of research laboratories from Rockefeller University as well as Harvard, Tufts, Case Western, Virginia, Washington, Oxford, and Cairo Universities, UNAM in Mexico, and the Weizmann Institute in Israel. I met extraordinary scientists (and, as it turned out, lifelong colleagues) at annual GND Network meetings held at the Woods Hole Marine Biological Laboratory. Prof. John David at Harvard was especially supportive of my taking on the problem of human hookworm infection.

I was especially eager to study hookworm infection because it was an enormous global health problem and yet at the time there were few if any laboratories attempting to apply modern molecular biology to its study. I remember coming across a paper while searching the stacks of the Rockefeller University library (Hotez 2014), which was written by Dr. Norman Stoll, a former Rockefeller Institute professor twenty years before (Stoll 1962). In it, he felt that hookworm was second in importance only to malaria because so many people were infected and the debilitating anemia they suffered due to the ability of adult hookworms to feed on human blood while attached to the intestine (Hotez et al. 2005). Human hookworm infection, caused by either *Necator americanus* or *Ancylostoma duodenale*—two closely related parasitic nematodes—is a leading cause of moderate and severe anemia in LMICs (Hotez et al. 2004). Because of their low underlying iron reserves, children and pregnant women represent two human populations who are considered especially susceptible to hookworm blood-feeding and blood loss.

The approach Tony and I discussed as a therapeutic or preventive strategy for hookworm infection resembled an idea proposed by Asa Chandler in the 1930s—to induce anti-enzyme antibodies (Hotez et al. 1987). In the case of hookworms, the idea would be to generate antibodies directed at key enzymes the adult hookworms were releasing at their site of attachment in the intestine (Hotez et al. 1987). This could be achieved by first discovering and isolating the major hookworm enzymes used by the parasite to digest blood, and then administering the enzymes as recombinant protein vaccines.

Together with Prof. Edward Reich who had discovered a role for proteases involved in plasminogen activation and anticoagulation (Unkeless et al. 1974), I began looking at fibrinogenolytic proteases released by hookworms. The first problem was obtaining adult hookworms for study. Ultimately, I found them through two sources. The first, was recovering adult dog hookworms of the genus *Ancylostoma caninum*, which were provided by the Auburn University College of Veterinary Medicine in Alabama. Next, I developed a close working relationship with Prof. Gerhard Schad at the University of Pennsylvania (Penn) School of Veterinary Medicine who was also working on animal models of hookworm infection (Schad 1991). Taking the Amtrak from Penn Station, New York, to 30th Street Station, Philadelphia, and then walking across the Drexel and Penn campuses to visit Prof. Schad at the Penn Vet School (38th and Spruce Streets) where we had long conversations both about hookworm and his philosophy of science is an especially fond memory of my MD-PhD student years. Prof. Schad became a lifelong friend and mentor.

In 1983, I published my first single-author paper with Tony on the discovery of a fibrinogen-degrading protease released by adult hookworms (Hotez and Cerami 1983). I was thrilled to have a paper published in the *Journal of Experimental Medicine*, which was one of the first important medical research journals in America, established at the Rockefeller Institute in 1905. After the paper was accepted, Tony told me that Prof. Maclyn McCarty from Rockefeller University was a reviewer on the paper. During the 1940s, together with Oswald Avery and Colin MacLeod, Dr. McCarty discovered that DNA comprised the genetic material of a cell. This finding was also published in the *Journal of Experimental Medicine* (Avery et al. 1944). Subsequently, Tony encouraged me to begin working with an extraordinary protein biochemist in the Laboratory of Medical Biochemistry, named Dr. Nguyen Le Trang, who taught me the art and science of column chromatography and protein purification. This led to a second paper on the protease (Hotez et al. 1985). I thought if we could clone and express the genes for hookworm blood-feeding enzymes, they could make potentially promising recombinant protein vaccines (Hotez et al. 1987) and began attempting to do this in the laboratory of Prof. Nina Agabian at UCSF, spending time in her unique laboratory established in Oakland, CA in collaboration with the U.S. Navy. Ultimately, this approach turned out to be a productive one, and (as will be described below) years later our research group accelerated two recombinant protein adult hookworm enzymes through clinical trials as part of a larger human hookworm vaccine initiative.

## Boston and the Massachusetts General Hospital

**Fig. 3:**
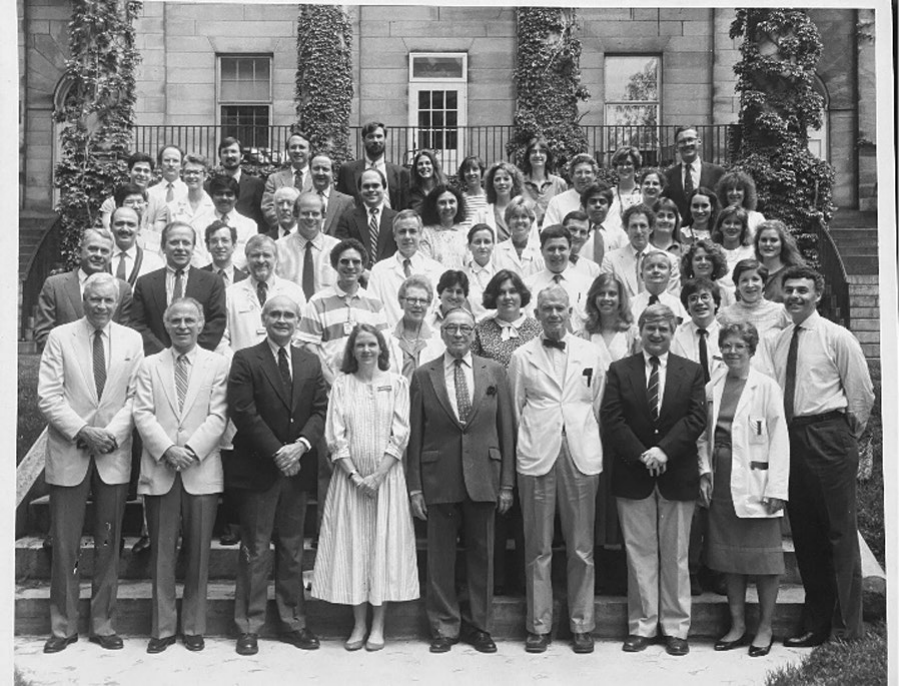
The Children’s Service, Massachusetts General Hospital, 1987–88. The author is in the second row on the right

After my doctoral dissertation and graduation from Rockefeller University for my PhD in 1986, I returned to Cornell and completed my MD degree. I enjoyed being on the wards, and felt that because of my passion for vaccines, the most appropriate clinical specialty might be pediatrics and pediatric infectious diseases. I then met Ann, who at the time was working at People Magazine in Manhattan and is now a mental health writer. We were soon living together and married the following year after I became a pediatrics intern and resident at Massachusetts General Hospital (“Mass General”). We lived in an old and somewhat dilapidated apartment in Beacon Hill across the street from the hospital but were very happy to be in Boston. The Children’s Services of the Mass General was one of the first specialty services devoted to the care of children in America and was known for its small size and intellectual rigor. The MassGeneral provided house officers with enormous latitude in decision making. In fact, the attending physicians at that time were known as “the visit” because the house officers really were in a decision-making role. I was grateful for my time spent there and I sometimes think it was at Mass General where I first learned how to be comfortable making tough decisions with important consequences. The Children’s Service was run by Dr. Donald Medearis Jr who was an inspirational role model for many of us who worked there (Fig. 3).

While I was at the MassGeneral, Dr. Medearis encouraged me to write a review on hookworm infection in children to keep up with the literature while still in house officer training and to get to know colleagues in the field of pediatric infectious diseases (Hotez 1989).

## Yale-China

After two years at Mass General, I returned to Yale to complete my pediatric infectious disease postdoctoral training, but also to continue working with Frank Richards. Frank had organized a new center for molecular parasitology supported by the MacArthur Foundation, and together with Department of Pediatrics, I did a simultaneous postdoctoral fellowship in both areas. The Section of Pediatric Infectious Diseases was headed by Prof. I. George Miller, a brilliant virologist interested in Epstein Barr Virus activation, and I developed lifelong friendships and associations with many of the attending pediatricians in that section including Drs. Eugene Shapiro, Warren Andiman, and Robert Baltimore. While in Frank’s lab, I continued working on adult hookworm blood-feeding but also turned my attention to the infective larval stages of hookworms that survive in the soil of endemic areas of LMICs. An important reason for studying infective larvae was based on studies conducted in the 1960s showing that living larvae damaged through ionizing radiation and administered by subcutaneous injection made an effective hookworm vaccine in dogs (Miller 1971). Although I did not think administering living irradiated hookworm larvae was feasible for humans in terms of population-wide immunizations, I thought that if I could find and isolate the antigenic molecules released by hookworm larvae during host entry through the skin, it was possible that their corresponding recombinant proteins could become promising vaccines. In this way, I hoped to shape a two-pronged approach to human hookworm vaccination—both larval and adult hookworm antigens, either combined or separately, with the adult hookworm antigens involved in parasite blood-feeding (Hotez et al.^1996^).

**Fig. 4:**
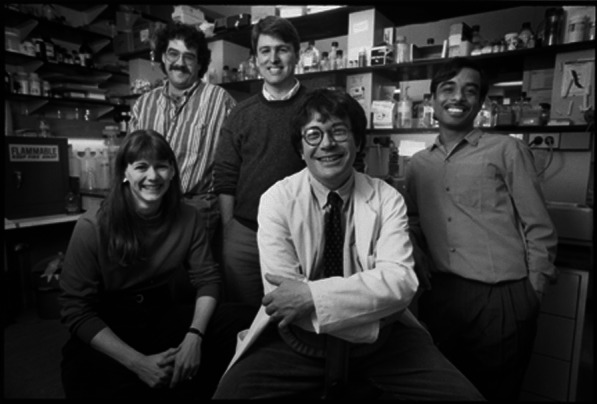
The Laboratory of Medical Helminthology, Yale University School of Medicine in the 1990s. From *Yale Medicine* with permission

Almost immediately after beginning in Frank’s laboratory, I found that hookworm larvae released two skin penetrating enzyme, a protease and hyaluronidase (Hotez et al. 1990, 1992). Then, upon recruiting Dr. John Hawdon (following his PhD with Gerhard Schad at Penn) as my first postdoctoral fellow, we found two more abundant hookworm antigens released by migrating hookworm larvae. These were highly cysteine-rich and stable proteins of unknown function at the time, and we named them *Ancylostoma* secreted proteins or ASPs (Hawdon et al. 1996, 1999). These CAP—an acronym of cysteine-rich secretory proteins, antigen 5, and pathogenesis related proteins—proteins have subsequently been found across multiple parasitic nematodes and are believed to play a role in the developmental biology of the parasite during or after larval host entry (Liu et al. 2023). I also brought Dr. Michael Cappello, an infectious disease postdoctoral fellow, to our laboratory who continued to investigate blood-feeding mechanisms of adult hookworms. Michael discovered a family of anticoagulants peptides that operated by interfering with mammalian blood factor Xa and other coagulation cascade enzymes (Cappello et al.^1995^). Dr. Kashinath Ghosh became another valued member of the laboratory. In time, I received independent funding from the NIH and private foundations to establish a modest-sized laboratory located in the major public health building at Yale and named it the Laboratory of Medical Helminthology (Hotez and Pritchard 1995) (Fig. 4).

**Fig. 5:**
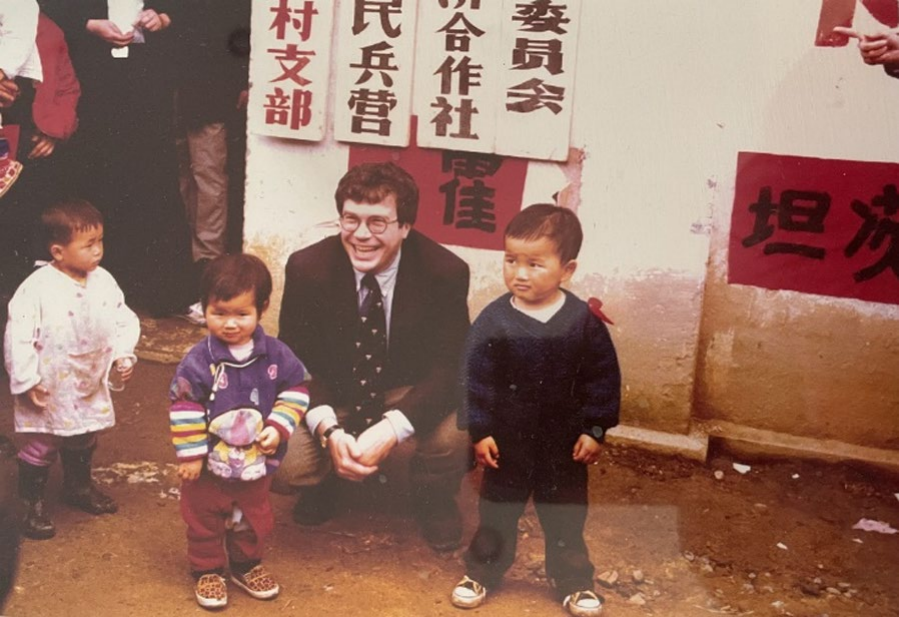
The author with children in rural Anhui Province, China where hookworm infection was widely endemic. From Washington Post https://www.washingtonpost.com/archive/politics/1998/05/27/chinas-new-hookworm-war/46bde149-087b-4692-b997-5b03e5d7f399/

At this time, I also began my clinical responsibilities as an attending pediatrician and pediatric infectious disease consultant at Yale-New Haven Children’s Hospital. However, another important aspect of my time at Yale, was a new collaboration with scientific and public health institutions in the People’s Republic of China. Yale University has had a long presence in China that began in 1901 and this continues today. Initially it was known as Yale-In-China and was focused on medical and nursing education and located in Changsha in Central China’s Hunan Province (Holden 1964). Shortly after I returned to Yale in the early 1990s, Frank Richards got very interested in expanding our university’s presence there and began collaborations with Fudan University in Shanghai. In parallel, I became interested in expanding the footprint of our Laboratory of Medical Helminthology because the Chinese Academy of Preventive Medicine (since renamed the Chinese CDC) had organized large-scale epidemiological studies conducted by parasitologists to find widespread parasitic infections among its population, especially hookworm and schistosomiasis across China’s impoverished rural interior (Hotez et al. 1997) (Fig. 5). Although this situation was rapidly changing due to rapid economic development in Eastern China cities, the rural areas in the interior had been left behind and suffered greatly from parasitic infections (Hotez 2002).

China has a long and tragic history with parasitic worms. Human hookworm disease and anemia was a major barrier to China’s economic development, as was schistosomiasis, which some scholars say thwarted Chairman Mao’s ambition to launch an amphibious assault on Taiwan following the Communist takeover of the mainland in 1949 (Hotez 2002, 2022a). Schistosomiasis is caused by a blood fluke that lives in the small veins draining the intestines or bladder and was found widely among both people and water buffalo inhabiting the agricultural areas along the tributaries of the Yangtze River. A close inspection of the marshlands along those tributaries reveals that it is teaming with *Oncomelania* snails that release the larval schistosomes into fresh water. These larvae known as cercariae have the ability to penetrate human skin to cause disease.

Together with colleagues at the Chinese Institute of Parasitic Diseases based in the old French Legation of Shanghai, and an international team that included the research groups of Profs. Donald McManus at the Queensland Institute of Medical Research (Australia), David Blair at James Cook University (Australia), and George Davis at the Academy of Natural Sciences in Philadelphia we obtained support from the NIH to look at the evolving human ecology of hookworm, schistosomiasis, and a parasitic lung fluke infection known as paragonomiasis. At that time there was particular interest in determining whether the new Three Gorges Dam being built on the Yangtze River to expand hydroelectric power for the region might inadvertently trigger an explosion in *Oncomelania* snail populations and therefore the re-emergence of schistosomiasis (Hotez et al. 1997; Hotez 2022a). Dam construction was previously shown to have disastrous consequences for promoting widespread schistosomiasis in Egypt and the Upper Volta region of West Africa (Hotez 2022a). Later we also obtained support from the China Medical Board (based in New York City) and involve colleagues from Peking Union Medical College. Previously, Peking Union Medical College had received generous support from the China Medical Board, which originally was a spinoff of the Rockefeller Foundation. Built around the same time as the major buildings of Rockefeller University, in my visits to Peking Union Medical College I was impressed how closely the architecture of both institutions resembled with other, except for the Chinese pagoda-styled roofs on the China side.

Our collaborations in China were highly productive in terms of generating research papers and expanding our knowledge about the ecology and epidemiology of parasitic disease. It was especially interesting to watch how economic development accelerated reductions in the prevalence of these infections, and vice versa, how parasite control fueled economic development (Hotez 2002). In China, I also met Dr. Bin Zhan who became a lifelong science partner and someone who came back with me to Yale where we worked closely together. Bin and I have been friends and scientific collaborators for 30 years. However, my major aspiration and goal to develop parasitic disease vaccines, beginning with a human hookworm vaccine had stalled. For that we would require more funding, laboratory space, and advice from experts with experience in developing vaccines. This required us to relocate and expand operations in Washington DC.

## Washington DC vaccine diplomacy: Brazil and beyond

The antigens discovered at the Yale Medical Helminthology Laboratory and from my Rockefeller University graduate work were essential but not sufficient (by themselves) to actually produce a human hookworm vaccine. Typically, discoveries made in academic laboratories are licensed to pharma companies or biotechs where they are transitioned into biopharmaceuticals made under current good manufacturing practices (cGMP) and accelerated through clinical trials under the auspices of a national regulatory authority, the US Food and Drug Administration (FDA) in the case of the United States. But for a human hookworm vaccine intended for use almost exclusively in LMICs—and for the very poorest individuals in those nations—there is not typically a pharma company or biotech willing to take this on due to an absence of any significant financial return.

One solution for this problem is to establish a PDP or product development partnership. These are non-profit organizations that use pharma or biotech industry practices to make the drugs, vaccines, or diagnostics that would not ordinarily be of interest to for-profit entities (Hotez et al. 2016). Two of the best-known PDPs are the Seattle-based PATH (formerly known as the Program for Appropriate Technology in Health) and DNDi (Drugs for Neglected Disease Initiative) headquartered in Geneva, Switzerland. While still at Yale I had met H.R. “Shep” Shepherd, an industrialist and entrepreneur, who became interested in vaccines and got to know Dr. Albert Sabin towards the end of his life. Shep persuaded Dr. Sabin and his wife Heloisa to allow the Sabin name for his new non-profit organization that was initially committed to vaccine advocacy but eventually became a PDP, with its initial focus on our human hookworm vaccine. The Sabin Vaccine Institute began in Connecticut (where I first met Shep while I was still at Yale) but then it re-established in Washington DC in association with the George Washington University (GWU) School of Medicine and Health Sciences. I left Yale to become Chair of the GWU Department of Microbiology (eventually renamed the Department of Microbiology, Immunology, and Tropical Medicine) while simultaneously serving as a senior scientist for the Sabin Vaccine Institute, now also based in Washington DC.

**Fig. 6:**
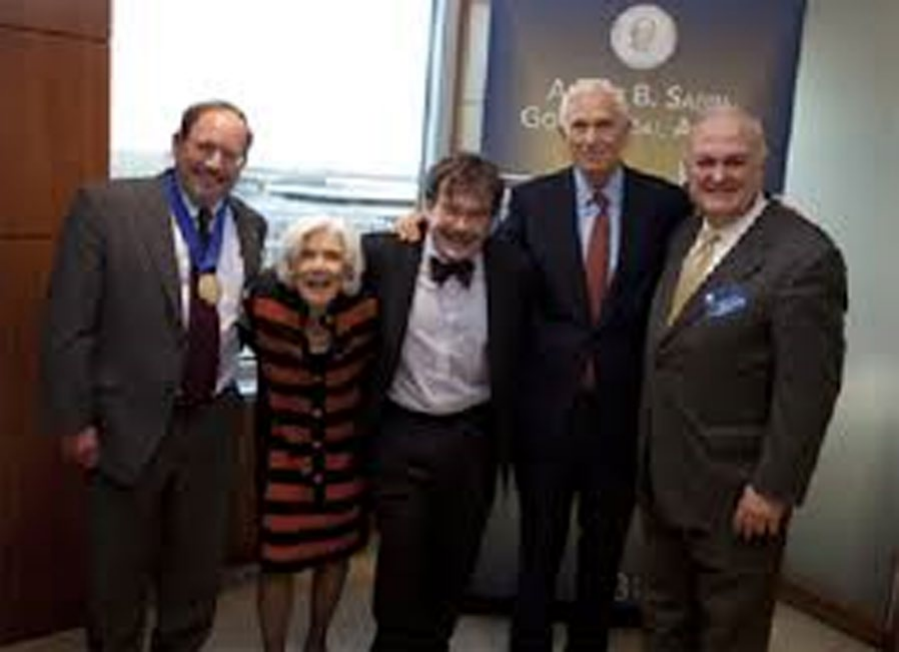
Meeting of the Sabin Vaccine Institute where the Sabin Gold Medal was awarded to Dr. John Clemens. From Left to Right: Dr. John Clemens, Heloisa Sabin, the Author, Mort Hyman, GWU President, Steven Knapp

Instrumental in this new initiative was Dr. Philip K. Russell, a retired Major General in the US Army and former head of Research & Development Command for the US Army Medical Research Institute of Infectious Diseases (USAMRIID) and Walter Reed Army Institute of Research (WRAIR), both located in the Washington DC area. Phil was an expert in tropical diseases and vaccine development, and he became an important mentor for me and our team of scientists, educating us on the steps necessary to actually make vaccines, rather than conduct pure academic research. Under his leadership our scientists at the Sabin Vaccine Institute and GWU organized a PDP devoted to parasitic disease vaccines. Phil had a passion for global health and vaccines, and he previously helped to create the Children’s Vaccine Initiative, a forerunner of Gavi, the Vaccine Alliance, for vaccinating the world’s children (Russell 1993). His advice was also sought after by a newly created Bill & Melinda Gates Foundation, and Dr. Russell was instrumental in working with us to secure initial Gates Foundation support to become a Washington DC-based PDP. After Shep retired from the Sabin Vaccine Institute, Mort Hyman from New York became the board chair (Fig. 6). Mort was highly influential in both business and political circles having been the chief executive officer (CEO) of a major shipping company, but also serving on boards of several academic health centers. Like Shep, Mort Hyman became an important mentor and friend.

For our PDP activities related to the hookworm vaccine we continued on a two-pronged approach producing both adult and larval hookworm antigens. We wound up cloning and doing scale-up protein expression on a number of hookworm larval antigens, with the *Na*-ASP-2 antigen cloned from *Necator americanus* and expressed in *Pichia* yeast (Goud et al. 2005) determined to be the most promising based on its protection in laboratory animal challenge models (Bethony et al. 2005) and findings suggesting that it was a key antigen responsible for the immunity observed in irradiated larval veterinary hookworm vaccines (Fujiwara et al. 2006). Work went into understanding the mechanisms of protection associated with *Na*-ASP-2, including solving the X-ray crystal structure of the antigen (Asojo et al. 2005). In a phase 1 “first in human” clinical trial conducted in Washington DC among normal volunteers the antigen when formulated on aluminum hydroxide (alhydrogel) was shown to be safe and immunogenic (Bethony et al. 2008), and we began a partnership with Instituto Butantan, one of Brazil’s two large vaccine producers (the other is FIOCRUZ-BioManguinhos) based in Sao Paulo for its industrial scale production. Unfortunately, the vaccine was shown to allergenic when phase 1 trials were repeated in a hookworm-endemic region of Brazil due to the fact that residents living there acquired some natural protection from IgE responses against this antigen, as a result of chronic exposure to soil-transmitted infective hookworm larvae. This was shown by Drs. David Diemert and Jeff Bethony, two senior scientists in our PDP based at GWU (Diemert et al. 2012). We had developed an exciting hookworm vaccine candidate, which was efficacious in laboratory animal models, and safe and immunogenic in human volunteers from non-endemic areas, but not a vaccine that could be advanced for hookworm-endemic LMICs.

**Fig. 7:**
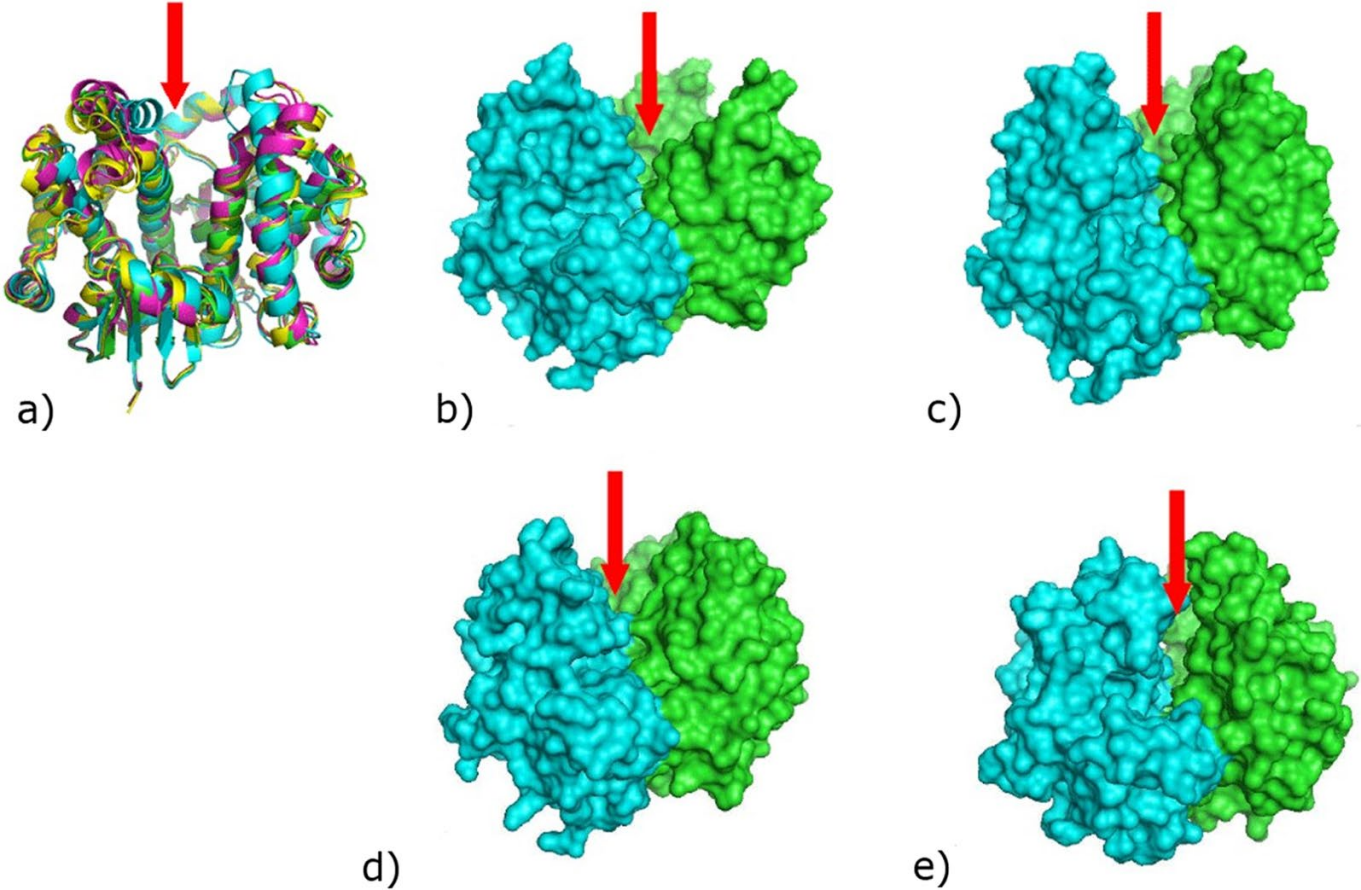
Comparison of GST dimers. **a** Superposition of GST dimers reveals that they are very similar, however, Nu class (*Na*-GST-1, magenta; *Na*-GST-2, gold; HpolGST, green) have a more accessible binding cavity than sigma class (HsGST, cyan). The path to the binding cavity is indicated by the red arrow. The surface plots of Nu class GSTs **b** HpolGST **c** *Na*-GST-1 **d** *Na*-GST-2 reveal larger access way to binding cavity than **e** sigma class GST (HsGST). Asojo et al. 2007 Reproduced from

Fortunately, we had better success with adult hookworm (*Necator americanus*) antigens involved in blood feeding, including *Na*-APR-1, an aspartic protease involved in hemoglobin digestion (Pearson et al. 2009), and *Na*-GST-1, a glutathione S-transferase involved in heme detoxification (Zhan et al. 2005, 2010). Both APR-1 and GST-1 were protective in animal challenge models (Xiao et al. 2008), while the *Na*-GST-1 molecule was especially exciting both because of its novel mechanism of action and its ability to produce it at scale using a yeast expression system. Our PDP found that hookworms produce a specialized GST-1 with the ability to bind heme, which is toxic for the parasite (Zhan et al. 2005, 2010). Moreover, *Na*-GST-1, like *Na*-ASP-2, could be scaled for production at high yields in a *Pichia* yeast expression system (Goud et al. 2012; Brelsford et al. 2017). This was important since industrial-scale yeast expression is widely used in LMICs for recombinant hepatitis B vaccine production, making it possible to adapt LMIC vaccine production methods for the human hookworm vaccine. As shown in Fig. 7, Dr. Oluwatoyin Asojo in our PDP solved the X-ray crystal structure of recombinant *Na*-GST-1 produced in yeast to find it belongs to a specific Nu class of glutathione S-transferases. Unlike more conventional glutathione S-transferases involved in peroxidation, Nu class glutathione S-transferases exhibit a large binding pocket with capacity of binding exogenous molecules such as heme (Asojo et al. 2007).

After optimization of *Na*-GST-1 for protein expression yield and purity (Curti et al. 2014), The antigen was shown to safe and immunogenic in human volunteers both in Washington DC and in Brazil (Diemert et al. 2017). Unlike the larval hookworm antigens, the adult hookworm enzymes did not appear to induce IgE in individuals naturally exposed to hookworm in endemic areas. *Na*-APR-1 also underwent scale-up production and clinical testing. However, our PDP could not successfully produce *Na*-APR-1 in yeast, but instead required an alternative tobacco expression system (Seid et al. 2015). In collaboration with a HOOKVAC vaccine consortium led by Dr. Remko van Leeuwen at the Amsterdam Institute of Global Health and Development (Netherlands) and the Centre de Recherches Médicales de Lambaréné, both antigens were shown to be safe and immunogenic in healthy volunteers living in Gabon (Mouwenda et al. 2021; Adegnika et al. 2021).

Demonstrating how these antigens, when formulated appropriately, might actually protect against human hookworm infection had to wait until we relocated to Texas. In the meantime, our PDP leveraged our infrastructure for a hookworm vaccine to also advance the development of a vaccine against intestinal schistosomiasis, a major cause of intestinal and hepatic illness on the African continent and in Brazil (Hotez et al. 2010). The lead antigen from *Schistosoma mansoni* and known as *Sm*-TSP-2, was discovered by Profs. Alex Loukas, Mark Pearson, and Jeff Bethony as part of our PDP through a combination of proteomics and immunonomics analyses, and studies confirming this antigen protected mice challenged with *S. mansoni* cercariae (Tran et al. 2006; 2010). Now, both Drs. Loukas and Pearson are independent investigators based at James Cook University in Australia. Our PDP scaled recombinant protein *Sm*-TSP-2 in *Pichia* yeast for production in yeast (Cheng et al. 2013; Curti et al. 2013), and this recombinant antigen own to be safe and immunogenic when formulated on alhydrogel in human volunteers, both in the United States and in Brazil (Keitel et al 2019; Diemert et al. 2023). Now this schistosomiasis vaccine has entered phase 2 clinical testing (Hotez et al. 2019).

Finally, while in Washington DC, I had the opportunity to become deeply involved with global health advocacy. During the early 2000s, there was a push to accelerate global health interventions as part of a new set of United Nations Millennium Development Goals (UN MDGs), including targeted interventions for HIV/AIDS and malaria. This led to the US Congress appropriating funds for both illnesses through PEPFAR (US President’s Emergency Plan for AIDS Relief) and PMI (US President’s Malaria Initiative), as well as a Global Fund to Fight AIDS, Tuberculosis, and Malaria (Hotez 2022a). However, there was no similar global push for parasitic infections such as hookworm and schistosomiasis. Working with two colleagues from the United Kingdom, Prof. David Molyneux at the Liverpool School of Tropical Medicine and Prof. Alan Fenwick at Imperial College London, as well as Dr. Lorenzo Savioli at the WHO, we rebranded a group of these chronic and debilitating parasitic infections as neglected tropical diseases or “NTDs” and explained to policymakers the opportunities for bundling low-cost mass deworming and other preventive treatment programs for hookworm and other intestinal helminth infections, schistosomiasis, lymphatic filariasis, onchocerciasis, and trachoma (Molyneux et al. 2005; Hotez et al. 2006; Hotez et al. 2007; Hotez et al. 2011; Hotez et al. 2019). Our group of “Three Musketeers”—Molyneux, Fenwick, Hotez—worked with policymakers in the US Congress, White House, and the UK Parliament to explain this opportunity and convince them of the importance of appropriating funds for NTDs as part of mission-critical overseas development assistance. Today, more than one billion people are treated annually for these conditions, in addition to treating conditions such as scabies and yaws (Hotez et al. 2019). These efforts are supported through $100 million annual appropriations from the US Agency for International Development as well as other public and private partners, such that global NTD control represents one of the world’s largest ongoing global public health control programs. These efforts also inspired me to launch *PLOS Neglected Tropical Diseases* as the first open access journal for these conditions (Hotez 2007).

## The Texas Children’s Hospital CVD and a National School of Tropical Medicine

**Fig. 8:**
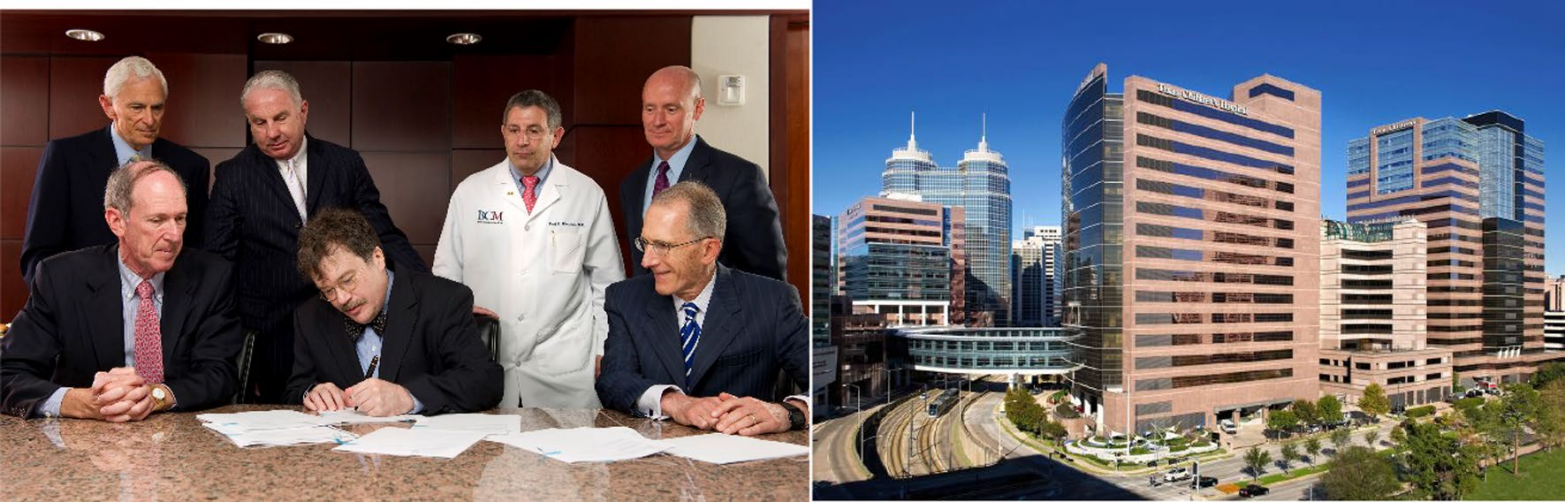
Left: Signing of the agreement to establish the National School of Tropical Medicine at Baylor College of Medicine and Texas Children’s Hospital CVD with the leadership of the Texas institutions, including Mark Wallace, the President and Chief Executive Officer of Texas Children’s Hospital, and Dr. Paul Klotman, the President of Baylor College of Medicine. Right: View of Texas Children’s Hospital including the Feigin Center to the far right, the location of the laboratories of the Texas Children’s Hospital CVD https://www.bcm.edu/about-us/affiliates/texas-childrens-hospital

For many years I have looked with admiration on the major tropical medicine schools in the United Kingdom, such as the Liverpool School of Tropical Medicine or the London School of Hygiene and Tropical Medicine, or those on the European continent, including the Swiss Tropical and Public Health Institute. Today, those institutions are leading cutting edge research for translational medicine in global health. I felt there was an opportunity to create a similar type of academic enterprise in North America, especially because of the emerging tropical infections in the Southern United States and Gulf Coast. Due to a confluence of poverty, climate change, urbanization, and human migrations, we noticed how this area of the country was seeing a sharp upswing in the frequency of NTDs including mosquito-transmitted virus infections such as dengue, chikungunya, West Nile virus and Zika virus infections, as well as Chagas disease, typhus, tickborne illnesses and other conditions (Hotez 2016; Hotez 2021; Hotez and LaBeaud 2023). The Texas Medical Center in Houston is the world’s largest medical center and perhaps the world’s first medical city, and it was attractive to create a new tropical medicine school there because of its Gulf Coast location and the fact that it was in one of North America’s fastest growing regions. That kind of growth and expansion create an openness and opportunities to build new types of institutions. Accordingly, in 2010, we signed an agreement to relocate both the research PDP arm of the Sabin Vaccine Institute and much of our laboratory at GWU to establish the National School of Tropical Medicine at Baylor College of Medicine and a new Texas Children’s Hospital Center for Vaccine Development (CVD). Establishing these institutions was made possible through the visionary leadership of Mark Wallace, the President and CEO of Texas Children’s Hospital and Dr. Paul Klotman, the President of Baylor College of Medicine, together with their respective board chairs at the time, Marc Shapiro and Gary Rosenthal (Fig. 8). Mort Hyman was an enthusiastic supporter of the relocation and expansion.

**Fig. 9:**
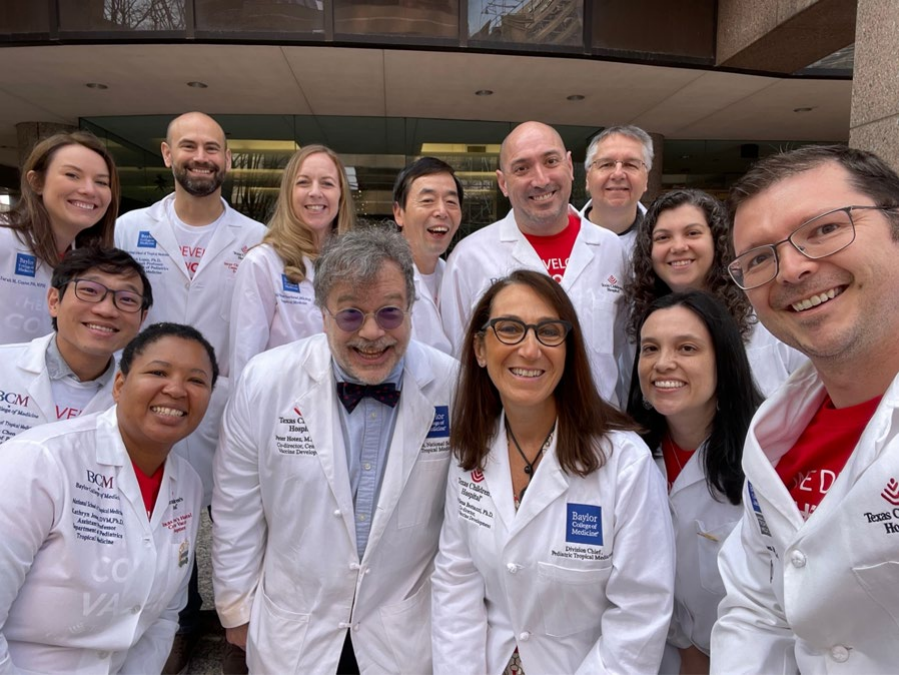
The faculty of the National School of Tropical Medicine. The author is standing in between Drs. Kathryn Jones (left) and Maria Elena Bottazzi (right)

Today the National School of Tropical Medicine has approximately 15 faculty conducting outbreak investigations and epidemiologic studies on emerging tropical infections, especially in the Western Hemisphere, with programs across Central America and tropical regions of South America, as well as in Asia, Africa, and the Middle East. Our faculty have also documented the emergence of NTDs and related tropical infections in Texas and the Gulf Coast. In addition, the Texas Children’s CVD is expanding its vaccine portfolio beyond hookworm and schistosomiasis vaccines, although we are very excited about recent information on the protective efficacy of our human hookworm vaccine in a human challenge model (Puchner et al. 2023). These include vaccines for leishmaniasis and Chagas disease. The Chagas disease vaccine is a therapeutic vaccine designed to delay or prevent the cardiac complications of chronic infection with *Trypanosoma cruzi,* the causative agent. Much of the experimental work is being led by Kathryn Jones DVM PhD in our PDP (Jones et al. 2022). The Chagas disease vaccine is now entering clinical trials through support of the Carlos Slim Foundation. Following Mort Hyman’s passing, our Texas Children’s CVD became independent of the Sabin Vaccine Institute in 2017. Today, I co-direct the Texas Children’s CVD with Dr. Maria Elena Bottazzi who has been a close science partner with me for the last two decades. Dr. Bottazzi is from Honduras and obtained her PhD at the University of Florida before conducting postdoctoral studies at the University of Pennsylvania and later GWU (Fig. 9).

Starting in 2012 our Texas Children’s CVD began partnering with the New York Blood Center, where two scientists, Drs. Shibo Jiang and Lanying Du, were exploring the receptor binding domain (RBD) of the spike protein as a potential coronavirus vaccine concept for new vaccines against severe acute respiratory syndrome (SARS) and Middle East Respiratory Syndrome (MERS) (Jiang et al. 2012). An attraction for the RBD versus the full-length spike protein were studies showing that it protected against challenge infections while minimizing antibody-dependent immune enhancement (Jiang et al. 2012). Like vaccines for parasitic infections or NTDs, vaccines for human coronavirus infections had similarly become orphaned and deprioritized by the big pharma companies. We had great success scaling up production of the SARS RPD in a *Pichia* yeast system (Chen et al. 2014; Chen et al. 2017) although the MERS RBD required mammalian cell expression. The RBD SARS vaccine was protective in a mouse SARS challenge model, which was work done in collaboration with Dr. Kent Tseng of the University of Texas Medical Branch in Galveston (Chen et al. 2020).

When the COVID-19 SARS-2 coronavirus sequence became available in January 2020, our Texas Children’s CVD was able to quickly pivot the coronavirus vaccine program to scaling up the production of the SARS-2 RBD in yeast and demonstrate its protection in laboratory animal models (Chen et al. 2021; Chen et al. 2022; Lee et al 2021; Pollet et al. 2021; Pollet et al. 2022), including a non-human primate challenge model in collaboration with the Emory National Primate Research Facility affiliated with Emory university (Pino et al. 2021). By this time, we began receiving requests from LMIC ministers of health and ministers of science for our COVID-19 vaccine technology, and upon doing our due diligence that the vaccine producers had a track record of working with the WHO to provide safe, and effective vaccines, we responded by providing the yeast production cell banks expressing the SARS-2 RBD antigen and detailed information about its scale-up expression. We did this without requesting additional patent protection from Baylor College of Medicine in order to facilitate rapid transfer of our technology and without attaching significant strings (Hotez 2023b).

Our efforts lead to the production and distribution of two vaccines. The first was Corbevax produced by Biological E in India, with almost 75 million doses administered to adolescent children for their primary immunization and millions more doses for adult boosters, and the second was IndoVac produced by BioFarma in Indonesia, which was also designated as a Halal vaccine (Hotez and Bottazzi 2022; Hotez et al. 2023). Both vaccines are comprised of the recombinant RBD domain of the SARS-2 spike protein adsorbed to aluminum hydroxide with a CpG oligonucleotide (Hotez and Bottazzi 2022; Hotez et al. 2023). The results of the clinical trials in India with Corbevax were published by Biological E (Thuluva et al. 2022a, b; Jaggaiahgari et al. 2022; Thuluva et al. 2023). In all, almost 100 million doses or more of the yeast-derived recombinant protein technology was administered to adults and children in India and Indonesia. This achievement provides evidence that it is possible to develop, produce, and deliver, a low-cost vaccine in a time of a pandemic without involvement of a multinational pharmaceutical company (Mahoney et al. 2023). Now, a low-cost XBB booster version of the Corbevax vaccine is also under development (Thimmiraju et al. 2023).

## Countering antivaccine activism

Beyond vaccine development, my scientific career gradually acquired a second and parallel pathway in public engagement. This aspect began when (as described above) I helped to champion the importance of addressing the NTDs in LMICs, working with the US Congress and other branches of the US Government, as well as with the UK Parliament (Hotez 2022a). It also continued when I began raising awareness about the NTDs in low-income areas of the Southern United States (Hotez 2008). Another major step in the public sector is when I agreed to expand our efforts in vaccine diplomacy. At the end of 2014 I was appointed by the Obama White House and its Office of Science and Technology Policy (OSTP), together with the U.S. State Department, as US Science Envoy for the Middle East and North Africa (MENA). The U.S. Science Envoy Program was established by President Barack Obama in 2009 when shortly after his inauguration he traveled to Cairo and announced at Cairo University a new engagement in international scientific cooperation with Muslim majority countries (Hotez 2021). During 2015 and 2016 I traveled extensively with the U.S. State Department and collaborated with American embassies in several nations in the MENA region to promote vaccine development. This included a meaningful capacity-building initiative with Saudi Arabia.

However, my greatest challenge in the public square has been countering a growing and increasingly audacious antivaccine lobby and movement. My involvement accelerated after the birth of youngest daughter Rachel, when she was diagnosed with autism and intellectual disabilities. At the time I was a junior faculty member at Yale University School of Medicine, and Rachel was diagnosed at the renowned Yale Child Study Center. At that time Ann’s parents, Don and Marcia Frifield, made frequent trips from their home in New Jersey to help us with Rachel. When Rachel was still very young girl, a paper was published in the *Lancet* claiming to show that 12 children who received their measles-mumps-rubella (MMR) vaccine progressed to a gastrointestinal condition associated with nonspecific colitis and intestinal lymphoid hyperplasia followed by developmental regression and pervasive developmental disorder or autism (Wakefield et al. 1998). Despite multiple epidemiological studies showing that there is no link between MMR vaccine and autism (and a retraction of the *Lancet* paper), the claims linking autism and childhood vaccinations persisted, eventually alleging it was thimerosal preservative, or spacing vaccines close together, or aluminum in vaccines that triggered neurodevelopmental degeneration and autism (Hotez 2018).

**Fig. 10:**
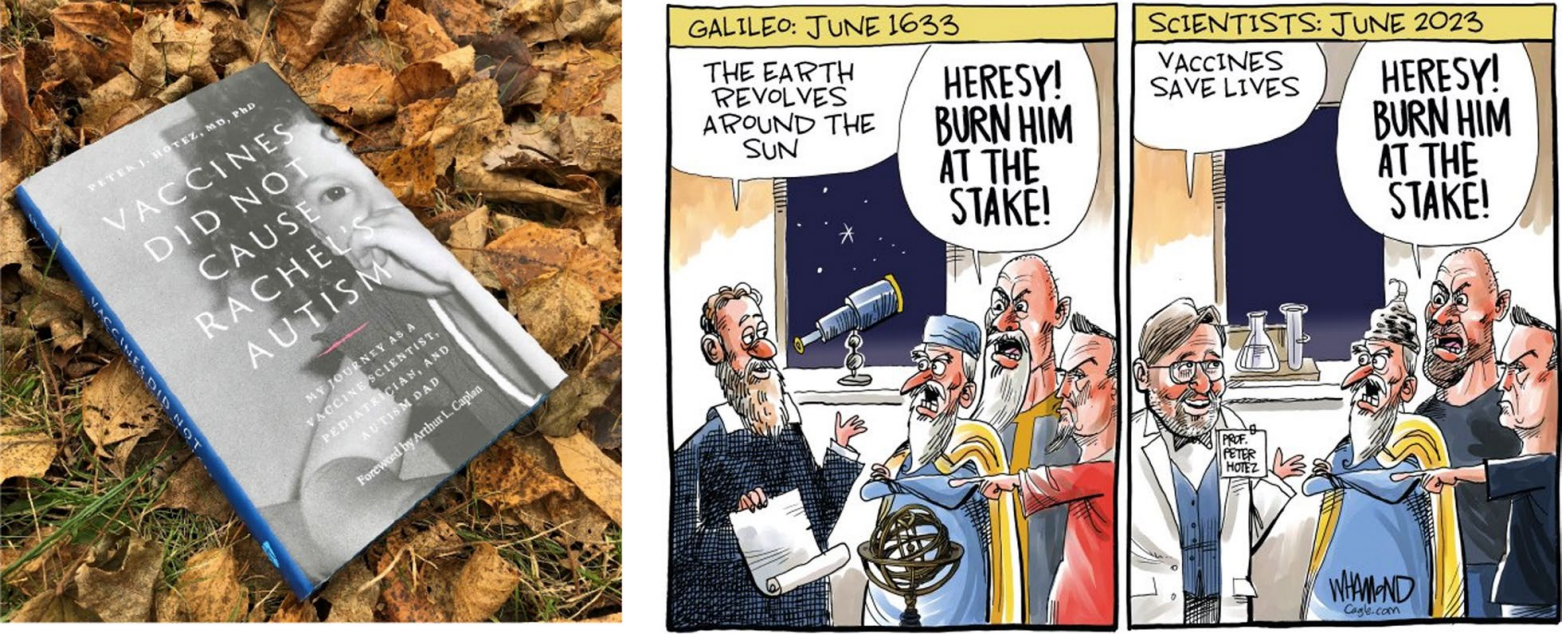
Left: The author’s book, Vaccines Did Not Cause Rachel’s Autism (Johns Hopkins University Press); Right: A 2023 political cartoon from Dave Whamond

As a pediatric vaccine scientist and a parent of a daughter with autism and intellectual disabilities I noticed there was a vacuum or absence of senior scientists or scientific organizations defending vaccines. In response I wrote *Vaccines Did Not Cause Rachel’s Autism*, a book which placed me front and center in the public defense of vaccines (Fig. 10).

I found this new public role meaningful although it became daunting and even scary at times, as antivaccine activists directed their online attacks against me through intimidating emails and efforts to discredit my science or me personally on social media. I also began to be stalked at meetings where I was scheduled to speak, or eventually at my home. These threats accelerated even further during the COVID pandemic when the antivaccine movement was adopted by a major political party in the United States and I became a target from members of the U.S. Congress and other elected officials, as well as a major cable news network linked to far-right extremism. In a subsequent book, *The Deadly Rise of Anti-science: A Scientist’s Warning* (Johns Hopkins University Press), I reported how 200,000 Americans needlessly died because they refused a COVID vaccine during the delta and BA.1 omicron COVID waves in 2021–22 (Hotez 2023a). In this new book I detail why so many Americans refused vaccines and how they became victims of a predatory and politically motivated antivaccine disinformation campaign (Hotez 2023a). The backlash to *The Deadly Rise of Anti-science* book accelerated the public attacks against me online or on cable news channels from elected officials, including at least two U.S. Senators, some members of the U.S. Congress, and two U.S. Presidential candidates, as well as several Fox News anchors. Even though I point out that my intention is to save lives and not to judge individual political leanings, my highlighting the unnecessary deaths from vaccine refusal is viewed as a *casus belli* from the far right and those with a political agenda.

Tragically, now the antivaccine movement is globalizing and spreading elsewhere in North and South America, Europe, and now even LMICs on the African continent (Hotez 2022b, 2023b). My concern is that our entire vaccine ecosystem is currently under threat, and we could see the widespread return of measles, polio, and other vaccine preventable diseases. Therefore, I view my public position to defend vaccines as one almost as important for saving lives as developing new vaccines.

## Concluding comments

My life in science took two parallel paths, the first was planned, deliberate, and strategic; the other was forged out of urgency and necessity. Regarding the first, since my student days I aspired to develop new vaccines for parasitic infections, later renamed NTDs, and now a human hookworm vaccine begun as a young MD-PhD student is now showing promise for safety and efficacy in advanced clinical development. Other NTD vaccines for Chagas disease and schistosomaisis have also advanced in clinical development, and most recently our similar approach proved to be useful to develop a low-cost COVID-19 vaccine technology that millions of people in LMICs. The second and unplanned aspect of my life was public engagement, initially to promote a pro-poor agenda in both LMICs and the Southern United States, and later to promote international vaccine and neglected disease diplomacy. With the birth of my daughter Rachel, my public activities focused on countering an aggressive, well-organized, financed, and politically charged antivaccine ecosystem. Both promoting vaccine science and countering vaccine anti-science required me to adopt a new skill set for in the areas of policy, advocacy, and communications. Along the way I learned how to write books with university presses. Both pursuing the science in the laboratory (and with it, the lab meetings, grants, and papers), and combating the anti-science in the public square have become a part of my academic and professional identity. In their own unique way, I find addressing the science and anti-science are both deeply meaningful and essential. We need both to prevent needless deaths and suffering from vaccine-preventable illnesses.

It is not always straightforward to take the lessons learned from my life in translational medicine and public engagement. Three potential take home points for young biomedical scientists include the following considerations:

First, do your best to create a roadmap or action plan for your biomedicine future. Often, I do whiteboarding exercises for young scientists and ask two questions (which often is the first time anyone has raised these issue): (1) What would success look like for you in 10–15 years such that you feel some sense of meaning and accomplishment, and (2) what problem in life do you wish to solve? Even if your plans change, creating such a roadmap is a useful exercise for maintaining some level of focus and conveying to your future mentors and colleagues a sense of enthusiasm and purpose. It also serves as a means to enlist others for sharing in your enthusiasm and successes.

Next, remember that academic medicine is hard work and academic health centers can be big, scary, and daunting enterprises. On the positive side, academic health centers are set up so you can make scientific breakthroughs or make lasting contributions to healthcare. Unfortunately, at times they can also be competitive when resources are limited. Success in the academic health center requires a sense of purpose as I have highlighted above, but also a degree of fortitude and perseverance. In pursuing a career in biomedical sciences there are more rejections and failures than successes. Scientific papers and grants get turned down, and many experiments don’t work. I sometimes say, “if science was easy, everyone would do it”. Find good and trusted colleagues at your institution and cultivate those relationships. For the last several years I have participated in a weekly Zoom meetings organized by Dr. Michael Osterholm, which includes Drs. Eric Topol, Margaret Hamburg, Penny Heaton, Bruce Gellin, and Ruth Berkelman. These have become some of my closest advisors, friends, and colleagues. Everyone experiences tough times in the academic health center, and you will need their support. Having moral courage is another key element, particularly now in this era of worsening anti-science aggression.

Finally, find joy in what you do, and try to emphasize activities you find meaningful and fulfilling. If everything you do in a week feels like a struggle or just provokes anxiety, think carefully and strategically to make professional changes. Don’t be shy about explaining your vision to your colleagues and superiors. Most senior leaders at academic health centers want you to succeed. I don’t particularly like the word “happy” since I often think of that as a childlike emotion, but if you feel a sense of fulfillment and part of the mix and doing important things, and take joy from that, this is the best of all. Remember too, that as you advance in your career, your priorities can shift. I became an author and book writer late in my scientific career—almost a decade after I became a full professor—and now I find writing to be one of my most impactful and worthwhile endeavors. It is never too late to learn new things. Along the way, take care of those you love and do your best to ensure that they too find paths to achieve their goals.

## Data Availability

Not applicable.
